# Application of Membrane Crystallization for Minerals’ Recovery from Produced Water

**DOI:** 10.3390/membranes5040772

**Published:** 2015-11-24

**Authors:** Aamer Ali, Cejna Anna Quist-Jensen, Francesca Macedonio, Enrico Drioli

**Affiliations:** 1 National Research Council—Institute on Membrane Technology (ITM–CNR), Via Pietro BUCCI, c/o University of Calabria, cubo 17C, Rende 87036, Italy; E-Mails: a.aamer@itm.cnr.it (A.A.); c.quistjensen@itm.cnr.it (C.A.Q.-J.); e.drioli@itm.cnr.it (E.D.); 2 Department of Environmental and Chemical Engineering, University of Calabria, Rende 87036, Italy; 3 WCU Energy Engineering Department, Hanyang University, Seoul 133-791, Korea; 4 Center of Excellence in Desalination Technology, King Abdulaziz University (KAU–CEDT), Jeddah 21589, Kingdom of Saudi Arabia

**Keywords:** membrane crystallization, produced water, membrane material, crystal characteristics

## Abstract

Produced water represents the largest wastewater stream from oil and gas production. Generally, its high salinity level restricts the treatment options. Membrane crystallization (MCr) is an emerging membrane process with the capability to extract simultaneously fresh water and valuable components from various streams. In the current study, the potential of MCr for produced water treatment and salt recovery was demonstrated. The experiments were carried out in lab scale and semi-pilot scale. The effect of thermal and hydrodynamic conditions on process performance and crystal characteristics were explored. Energy dispersive X-ray (EDX) and X-ray diffraction (XRD) analyses confirmed that the recovered crystals are sodium chloride with very high purity (>99.9%), also indicated by the cubic structure observed by microscopy and SEM (scanning electron microscopy) analysis. It was demonstrated experimentally that at recovery factor of 37%, 16.4 kg NaCl per cubic meter of produced water can be recovered*.* Anti-scaling surface morphological features of membranes were also identified. In general, the study provides a new perspective of isolation of valuable constituents from produced water that, otherwise, is considered as a nuisance.

## 1. Introduction

Increasing demand of oil and gas has been observed despite the emphasis on using alternative energy resources. However, the oil and gas exploitation process produces huge volumes of high salinity wastewater known as produced water [1]. High concentrations of different metals present in produced water pose serious environmental threats, and, in certain cases, proper management of produced water governs the decision of further oil and gas recovery from a reservoir. As a consequence, a proper treatment of produced water can solve the environmental concerns and can be beneficial in bridging the gap between demand and availability of freshwater in water scarcity regions where generally oil and gas reservoirs are located. Moreover, abundance of different minerals present in produced water offers new perspective to recover valuable components from this wastewater stream. State-of-the-art treatment remedies for produced water include different chemical, biological and physical procedures [2]. However, conventional treatment methods have certain limitations including the usage of toxic chemicals, high cost of treatment, large footprint, long retention times and the creation of secondary pollution [1,2,3,4]. Moreover, new stringent environmental regulations put emphasis on more effective treatments, underlying the importance of adopting more efficient treatment options. Due to these reasons, recently membrane based treatments have been tried widely for produced water treatment [5,6,7,8,9,10]. Membrane distillation (MD) is an emerging process based on temperature gradient created across a microporous hydrophobic membrane. The process owns several advantages over the state-of-the-art techniques including the potential to use waste grade energy, ability to concentrate the solutions to their saturation level and theoretically complete rejection of all non-volatiles. These features make MD particularly attractive for processing produced water because of the energy level [11,12]. Technical feasibility of MD to treat produced water was confirmed by several recent studies [13,14,15]. The ability of MD to treat the solution beyond the saturation level has been well exploited in membrane crystallization (MCr). Unlike conventional crystallizers, in MCr a well-controlled nucleation and crystal growth are achieved through uniform evaporation rate through the pores of the membrane [16]. Therefore, MCr produces crystals of much higher quality in terms of purity and size distribution with respect to conventional industrial crystallizers. The potential of MCr to recover crystals and to target specific polymorphic salt structures from various concentrated solution was explored in various studies [17,18,19]. Application of MCr for produced water treatment provides the opportunity to simultaneously extract fresh water and minerals by utilizing the low grade energy associated with produced water during production process.

Successful application of MCr for crystallization from produced water is, however, strongly related with the availability of suitable membranes and understanding of associated heat and mass transport phenomena. Membrane material, morphology and surface characteristics play a crucial role in dictating process performance and stability [20,21]. Besides that, utilized membranes may also affect the crystallization kinetic, polymorph and crystal characteristics as demonstrated in studies addressing the crystallization of protein molecules [19]. The objective of the current study is to investigate the MCr potentialities for recovery of minerals and freshwater from microfiltered oilfield produced water. The effect of process variables including feed inlet temperature, feed flow rate and membrane material on transport phenomena and crystal characteristics has been scrutinized.

## 2. Materials and Methods

### 2.1. Membrane Used

Commercial polypropylene (PP) and lab made polyvinylidene fluoride (PVDF) hollow fiber membranes were used in the experiments. For investigation at semi-pilot scale, two commercial PP modules from Microdyn Nadir connected in parallel were used. Each module contained 40 fibers with 0.1 m^2^ surface area. For lab scale small plants, modules prepared in the laboratory with commercial PP and lab-made PVDF hollow fiber membranes were used. Synthesis of PVDF membranes used has been reported elsewhere [22]. The main properties of the membranes and modules applied are provided in Table 1.

**Table 1:** Main properties of membrane applied in current study.

Fiber Type	Thickness	Emod	Rm	Break	W	PMI Bubble Point	PMI Pore Size	Porosity	Membrane Area
	(mm)	N/mm^2^	N/mm^2^	%	Nm	(bar)	(µm)	(%)	(m^2^)
PP lab-made module	0.45	103.75	4.16	174.40	1.11	0.76	0.2	73	0.0056
PP Commercial modules	0.45	–	–	–	–	–	0.2	73	0.2
PVDF	0.40	65.76	3.86	259.95	0.71	0.87	0.23	80.77	0.0021

### 2.2. Feed Composition

Produced water for the current investigations was provided by Kuwait Institute of Scientific Research (KISR). The main characteristics of this water can be found in [23]. The water contains 248 g/L of TDS and traces of volatile compounds. Ionic analysis carried out by ionic chromatograph (Metrohm, CH-9101) is shown in Table 2. Total organic carbon (TOC) analysis was performed according to the detailed procedure described elsewhere [23]. In membrane crystallization, calcium is often removed by chemical treatment to avoid the undesired scaling phenomena [24]. Nevertheless, in this study, the produced water was not treated chemically prior to MD and MCr in order to evaluate the feasibility of direct treatment.

**Table 2:** Main properties of produced water used.

Property	Value	
TDS	248,000	
Conductivity (mS/cm)	228.2	
pH	6.15	
TOC	18.10	
TC	40.72	
Sodium Na	76,646	
Calcium Ca	6065	
Magnesium Mg	8361	
Potassium K	1396
Chloride Cl	144,057
Phosphate	1055
Sulphate SO_4_	1213
Nitrate NO_3_	613
Floride F	472

Note: All concentrations are in ppm.

### 2.3. Membrane Crystallization Tests

**Figure 1:**
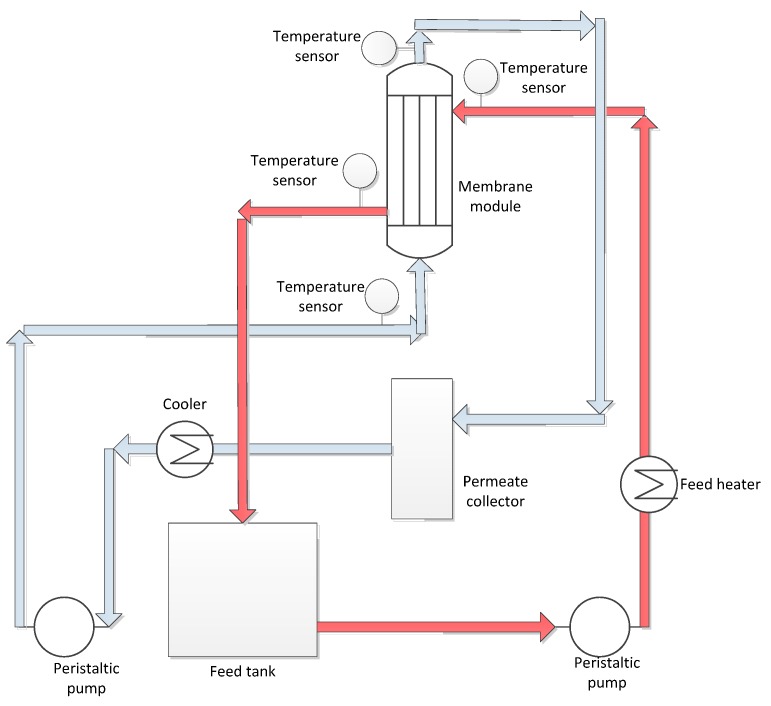
Schematic diagram of the set-up used for MCr.

To test the initial technical feasibility of MCr for simultaneous recovery of water and salt crystals from produced water, experimentation was carried out by using small scale membrane modules. MCr tests were performed at feed inlet temperatures of 35 °C, 45 °C and 55 °C for each membrane while the permeate temperature was kept constant at 10 °C. For these temperatures, feed and permeate flow rates were adjusted at 150 and 70 mL/min, respectively. The effect of feed flow rate was inspected by changing the feed flow rate from 150 to 250 mL/min with an interval of 50 mL/min each. In order to avoid blockage of fibers due to possible scaling, outside-in configuration was applied. After confirming the technical feasibility of the process at small scale, the experimentation was extended at semi-pilot scale by using commercial PP modules from Microdyn Nadir at feed and permeate inlet temperatures of 40 °C and 15 °C, respectively. The quality of distillate was analyzed after regular interval by monitoring its conductivity. Figure 1 shows the set-up description used for experimentation at both scales.

### 2.4. Crystals Characterization

The obtained crystals were analyzed visually by using transmitted optical microscope (ZEISS model Axiovert 25). The images were recorded by applying a video camera model VISIOSCOPE Modular System equipped with optical head (10-100×). The recorded images were analyzed by using Image J software version 1.48 V from Wayne Rasband, National Institute of Health, USA. Crystal growth and kinetic were analyzed by taking the sample after every 30 min. The obtained images were applied to calculate population density, growth rate, average crystal size, crystal size distribution and coefficient of variation (CV) at different stages of experimentation for all the conditions analyzed.

CV was estimated through Equation (1), whereas growth and nucleation rate were estimated on the basis of the Randolph-Larson model (Equations (2) and (3), respectively):

(1) $$CV=\frac{F_{84\%}-F_{16\%}}{2\cdot F_{50\%}}$$

(2) $$\ln(n)=\frac{-L}{Gt}+\ln(n^{0})$$

(3) $$B^{0}=n^{0}G$$

where *F* is the cumulative percent function given by the crystal length at the indicated percentage; *n* is the crystal population density; *L* is crystal size; *G* is growth rate; *t* is retention time and *n*^0^ is population density at *L* equal to zero.

### 2.5. Heat and Mass Transfer Analysis

During the direct contact membrane distillation (DCMD) process, heat transfer from the feed side takes place via conduction through membrane and convection of vapors across the membrane, giving rise to thermal polarization. Heat and mass transport analysis was applied to explore the resistance to heat and mass transfer and to simulate the experimental flux.

Based on surface temperatures and membrane characteristic parameter *B*, theoretical flux can be calculated by using the following relationship:

(4) $$J=B\left(P_{fm}-P_{pm}\right)$$

where *P_fm_* and *P_pm_* are the vapor pressure corresponding to interfacial temperatures at feed (*T_fm_*) and permeate side (*T_pm_*), respectively. Dusty gas model has been widely applied to calculate the theoretical value of *B* [25,26]. According to the model, mass transfer through membrane pores can be expressed by the Knudsen diffusion model, molecular diffusion model or transition model. Membrane pore size and mean free path of vapors dictate the suitability of the applied model. In the current study, the mean free path of water vapors (~0.13 micron) is very close to the mean pore size of membranes applied favoring the application of transition model. According to this model, the membrane characteristics of parameter *B* can be calculated by using the following relationship:

(5) $$B={\left[\frac{3\tau \delta_{m}}{2\varepsilon r}{\left(\frac{\pi RT}{8M}\right)}^{1/2}+\frac{\tau \delta_{m}}{\varepsilon }\frac{Pa}{PD}\frac{RT}{M}\right]}^{-1}$$

where *τ*, *δ_m_*, *ɛ*, *r* and *M* are tortuosity factor, membrane thickness, porosity, average pore size of membrane and molecular weight of water. Calculation of temperatures at interfaces requires the knowledge of corresponding heat transfer coefficients. Various correlations for heat transfer coefficients were proposed in literature [27,28,29,30]. The appropriate heat transfer coefficient was calculated according to the procedure explained elsewhere [31]. In the current study, the following correlation was found to be the most suitable to simulate the process [29]

(6) Nu=4.36+0.036RePr(D/L)1+0.0011(RePr(D/L)0.8

On the basis of *Nu*, heat transfer coefficient can be calculated by using the following formula:

(7) $$h=\frac{NuK}{D}$$

Given the fact that composition of produced water comprises mainly NaCl (Table 2), the calculation procedure was simplified by assuming the properties of produced water equivalent to that of NaCl at identical concentration and at the same temperature conditions.

Knowledge of heat transfer coefficients allows calculating the corresponding *T_fm_* and *T_pm_* according to the following correlations:

(8) Tfm=Tf−(Tf−Tp)1hf1hv+hc+1hp+1hf

(9) Tpm=Tp+(Tf−Tp)1hp1hv+hc+1hp+1hf

where *h_v_*, *h_c_* and $\Delta {Η}_{\text{v}}$ represent vapor heat transfer coefficient, membrane heat transfer coefficient and vapor enthalpy at average membrane temperature and can be calculated by Equations (10)–(12).

(10) $${\text{h}}_{\text{v}}\text{=}\frac{\text{J}\Delta {Η}_{\text{v}}}{T_{fm}-T_{pm}}$$

(11) $$h_{c}=\frac{k_{m}}{\delta }$$

(12) $$\Delta {Η}_{\text{v}}\text{=1.7535}\left(\frac{{\text{T}}_{\text{fm}}{\text{+T}}_{\text{pm}}}{\text{2}}\right)\text{+2024.3}$$

Vapor pressure at the membrane surfaces can be calculated by using the Antoine’s equation.

(13) $$P=\exp\left(\text{23.238-}\frac{\text{3841}}{\text{T-41}}\right)$$

In the MD process applied to a solution, major resistances to mass transfer can be associated with boundary layers, membrane and solution concentration. The last two are intrinsic properties of the membrane and solution applied, respectively, while the former depend mainly upon the hydrodynamic and thermal conditions applied. Addition of solute into a solvent suppresses the vapor pressure of the solution relative to pure solvent. The reduction in vapor pressure can be estimated by using the following correlation proposed by Yun *et al.* [32]

(14) $$P_{s}=P^{o}(1-x)(1-0.5x-x^{2})$$

Based on solution concentration and membrane surface temperatures, resistances to mass transfer offered by feed and permeate side boundary layers (*R_f_* and *R_p_*, respectively), membrane (*R_m_*) and solution concentration (*R_c_*) can be determined by the following correlations:

(15) $$R_{f}=\frac{P_{f}-P_{fm}}{J}$$

(16) $$R_{p}=\frac{P_{pm}-P_{p}}{J}$$

(17) $$R_{c}=\frac{P_{fm)s}-P_{fm)w}}{J}$$

(18) $$R_{m}=\frac{P_{fm)s}-P_{pm}}{J}$$

## 3. Results and Discussion

### 3.1. Flux and Associated Resistances

**Figure 2:**
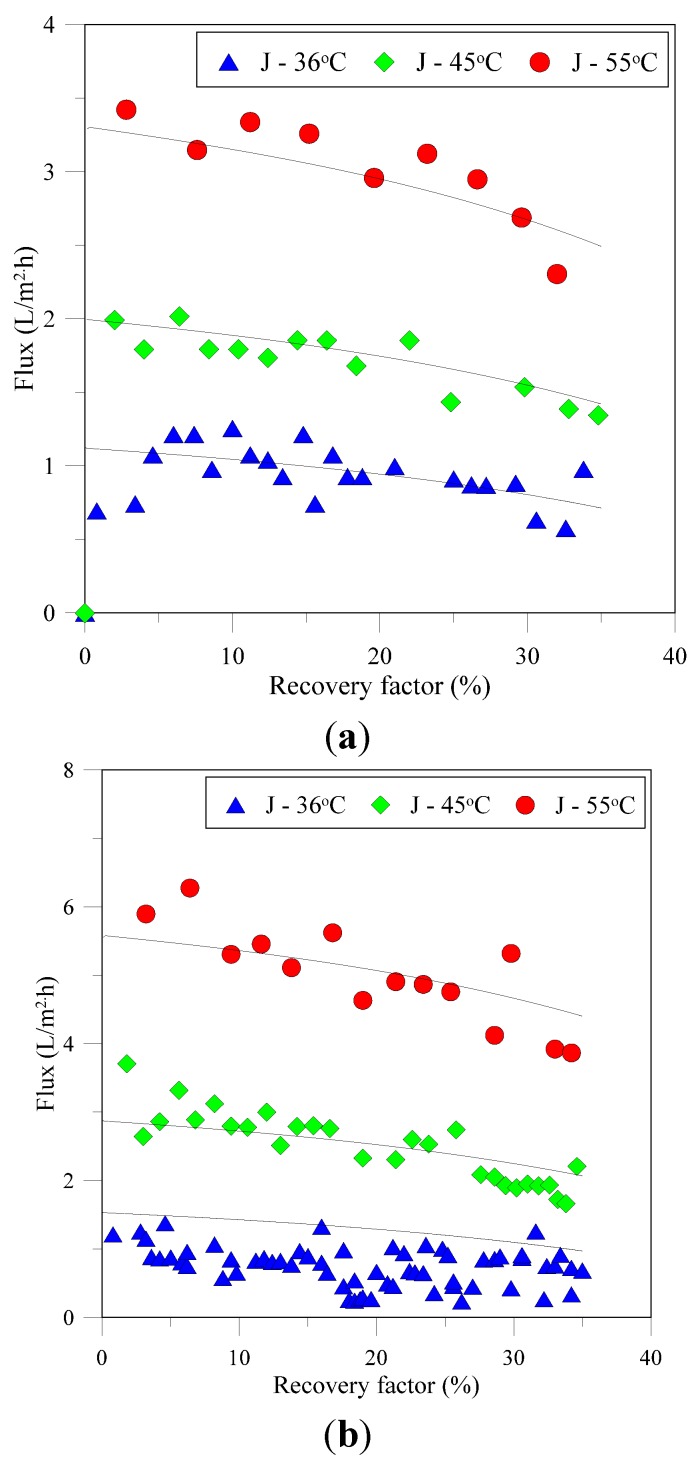

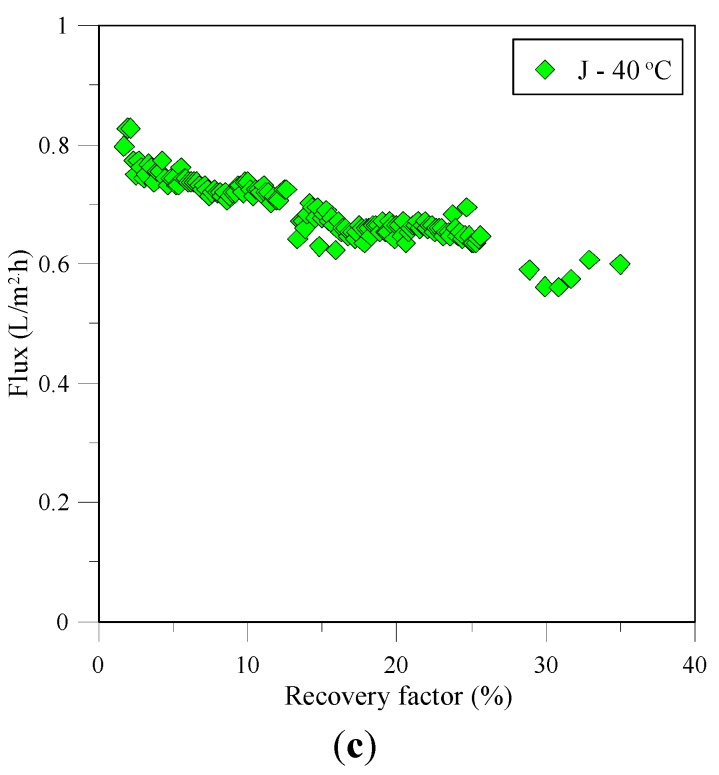
Discrete flux and recovery factors obtained at various thermal conditions for PP and PVDF membranes. (**a**) PP lab made modules (**b**) Lab made PVDF based modules (**c**) Commercial PP modules. The lines show the theoretical flux.

Discrete flux as a function of the recovery factor for PP (lab made and commercial modules) and PVDF membranes under various feed temperatures is shown in Figure 2. Theoretical flux calculated by taking into account concentration effect on solution properties is also provided in the same figure. A good agreement between experimental and theoretical flux is evident under all the conditions investigated for both membranes. The decrease in flux with experimental time in both cases can be attributed mainly to increase in solution concentration that decreases the vapor pressure of solution as evident from Equation (14). It can be noticed from the figure that flux for PP membrane increases from ~1 L/m^2^h to ~3.4 L/m^2^h when feed inlet temperature is raised from 35 to 55 °C. The same trend was observed for PVDF membrane, though the flux is higher in this case at all thermal conditions applied. This observation is coherent with the acknowledgement of exponential increase of vapor pressure and associated flux with feed temperature provided in literature [33,34]. The effect of concentration polarization can be neglected due to very low trans-membrane flux obtained in this investigation. At all temperatures, the crystals appear in the solution at similar recovery factor of ~33%, although induction time decreases at higher temperature as supersaturation level is reached faster. It is well acknowledged that the solubility of NaCl increases marginally by increasing the temperature therefore, the crystals are yielded at approximately same recovery factor even if the solution temperatures are different. Experimentation was carried out to achieve equal recovery factor (~37%) in both cases. The crystals were simultaneously recovered from the solution and dried in oven at 45 °C overnight and weighed hereafter. The calculations show that 16.4 kg NaCl per cubic meter of produced water are recovered at recovery factor (RF) 37%. Indeed, the amount of recovered salts will be high at higher recovery factors. Lower flux observed in case of commercial PP modules can be attributed with their higher length [34,35]. Due to transfer of vapors from feed to permeate side and direct contact of two faces of the membrane with hot feed and cold permeate, the temperature gradient along the fiber length decreases and results in reduction in average flux.

**Figure 3:**
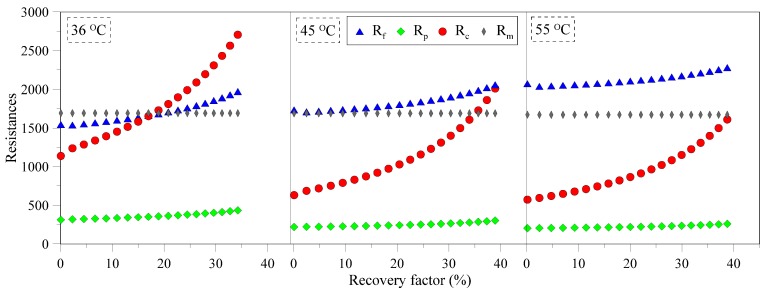
Various resistances to mass transfer at different temperatures as function of solution concentration.

Resistances to vapor transfer offered by membrane, boundary layers and solution as a function of RF can be found in Figure 3. Under the hydrodynamic and thermal conditions used in the current investigation, the feed side boundary layer and membrane itself offer the major resistance to mass transfer at relatively low solution concentration. *R_p_* and *R_m_* remain relatively insensitive towards increase in RF while *R_f_* shows weak dependence on RF. With increase in RF, concentration of solution increases and affects solution density and viscosity that affect hydrodynamic conditions within the module. Resulting poor hydrodynamic conditions may partly contribute in flux decay of the system. It is well established that thermal polarization is less severe at low feed temperatures [33,36], therefore, *R_f_* increases with increase in feed temperature as illustrated in Figure 3. *R_c_* increases rapidly after a certain RF (~20%) and dominantly controls the mass transfer for MCr systems operating at low feed temperatures.

In MD, a significant amount of heat is lost due to conduction taking place across the membrane due to direct exposure of membrane faces with streams at two different temperatures. Thermal efficiency (TE) defined by Equation (19) is a measure of heat utilized to vaporize the product to total heat transfer across the membrane.

(19) $$TE=\frac{J\lambda }{J\lambda +\frac{K_{m}}{\delta }\left(T_{fm}-T_{pm}\right)}$$

**Figure 4:**
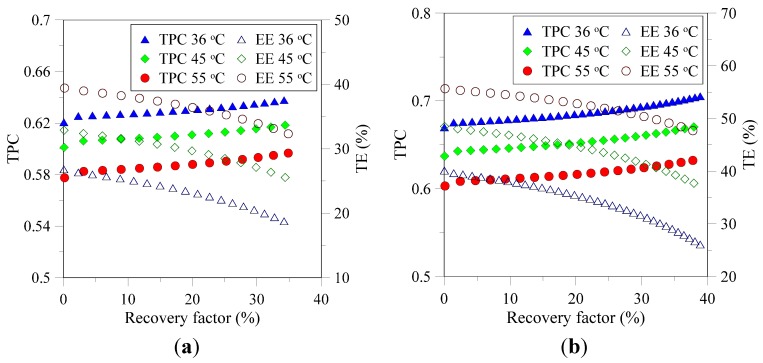
TPC and TE of PP (**a**) and PVDF (**b**) membranes as function of RF at various temperatures.

TE can be tuned by appropriate selection of membrane features and operating conditions applied so that the flux of the system is the maximum. Figure 4 shows that TE for the PVDF membrane is slightly higher than the PP membrane under all the conditions investigated. The observed trend can be attributed to high overall thermal conductivity of PP membrane which is a function of membrane material, its thickness and porosity [25,37]. Membranes synthesized with less conductive material and having high thickness and overall porosity display less thermal conduction losses. High TE of PVDF membrane observed in current study can be associated with its high porosity and inherently lower thermal conductivity of PVDF material. Figure 4 also reveals that TE decreases with increase in RF. As indicated in Figure 2, flux for both PP and PVDF membranes decreases with increase in RF and adversely affects TE.

Dependence of temperature polarization coefficient (TPC) on feed temperature and concentration for two studied membranes is illustrated in the same figure. It can be noted that TPC increases slightly with increase in solution concentration at all the conditions for both types of membranes. The trend is in agreement with previous studies where a similar increase was observed [38]. Two competing phenomena influence TPC: increase in solution concentration enhances the resistance to heat transfer (Figure 3) and at the same time reduces the flux of the system. The former phenomenon tends to lower TPC while the later reduces the convective heat flux through the membrane lowering the temperature gradient between bulk feed and membrane surface. The later phenomenon prevails the overall effect and contributes in overall observed increase in TPC. It can also be noted that increase in TPC with concentration is more prominent at higher feed temperatures.

### 3.2. Characterization of Recovered Crystals

**Figure 5:**
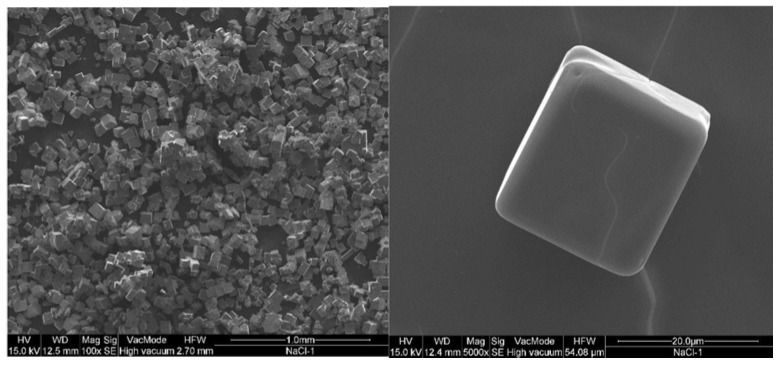
SEM images of the crystals precipitated from produced water (**a**) Area of crystal sample—magnification: 100×; (**b**) Single crystal—magnification: 5000×.

**Figure 6:**
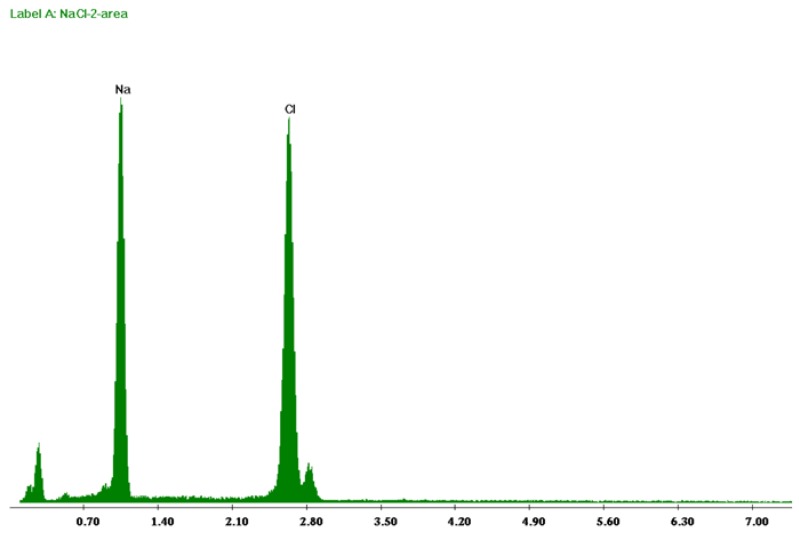
EDX spectra obtained for the crystals precipitated from produced water.

**Figure 7:**
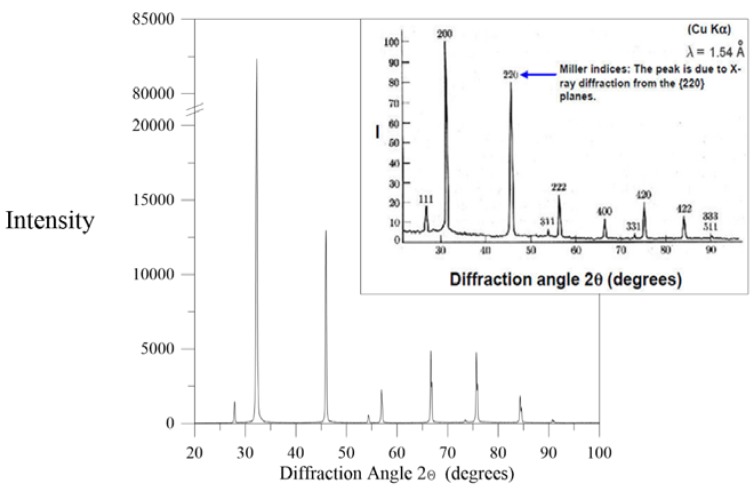
XRD spectra obtained from the recovered crystals from produced water. Spectra of NaCl from literature has been shown in inset.

In the current study, precipitation from produced water occurred around a recovery factor of 33%. The recovered crystals were analyzed with scanning electron microscopy (SEM), energy dispersive X-ray (EDX) and X-ray diffractometer (XRD). SEM images shown in Figure 5 at different magnifications illustrate a cubic structure. EDX spectra (Figure 6) clearly show that only sodium chloride without any impurities is crystallized. This analysis was confirmed by XRD (Figure 7) where the sample of crystals recovered from produced water shows the same peaks as for XRD spectra of NaCl from literature shown in inset of Figure 7. The recovered crystals were separated from the solution and were dried to estimate experimentally the quantitative potential of crystal recovery per unit volume of the feed. The weight of crystals separated from small and semi-pilot plant showed that 16.4 kg of high quality crystals can be recovered per cubic meter of produced water at water recovery factors of 37%.

**Figure 8:**
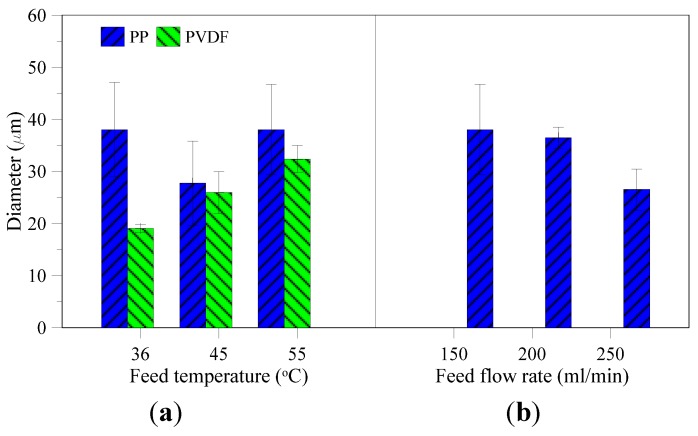
Mean diameter of the produced crystals at different feed temperatures and feed flow rates ((**a**,**b**) respectively).

**Figure 9:**
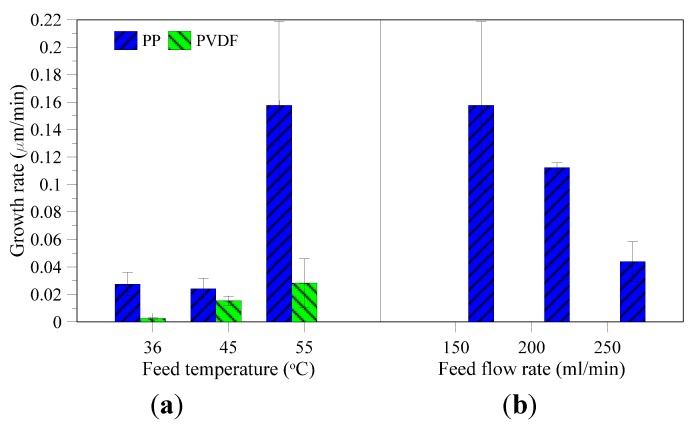
Growth rate of the produced crystals at different feed temperatures and feed flow rates ((**a**,**b**) respectively).

Once the first crystals were observed, a sample of mother liquid containing crystals was analyzed by optical microscope. Mean diameters of recovered crystals using PVDF membrane increase with increasing temperature (Figure 8a) due to the improvement of trans-membrane flux and hereby supersaturation gradient, which is the driving force for crystal growth. However, mean diameters of the produced crystals by using PP membrane at different feed temperatures do not show any clear trend. Growth rate shown in Figure 9a has the same trend as mean diameter and its value at constant feed flow rate is higher for the highest temperature, which is explained by the higher supersaturation ratio caused by the higher flux at 55 °C.

Comparing the PVDF and PP membranes, the growth rate by using the PVDF membrane is much lower (Figure 9a). The reason is the difference in membrane surface area between the two modules, *i.e.*, 0.0021 m^2^ for PVDF and 0.0056 m^2^ for PP, thus the same concentration factor can be reached sooner using PP membrane, and, therefore, the growth rate increases.

Besides supersaturation gradients, mean diameter and crystal growth are also influenced by feed flow rates, due to a control in the transfer of material from solution to crystal interface (diffusion) and by organization of material from the interface into the crystal lattice (integration) [39,40]. Either the diffusion or integration is the rate limiting step. If mean diameter and crystal growth increase with increasing feed flow rate, the diffusion step is rate limiting. Likewise, if mean diameter and crystal growth is suppressed at higher feed flow rates, it is the integration of material into crystal lattice which is the rate determining step. In crystallization from produced water, the average mean diameter and growth rate decrease with increase in feed flow rate (Figure 8b and Figure 9b). Therefore, the limiting step of crystal growth is the integration step under the given operative conditions.

**Figure 10:**
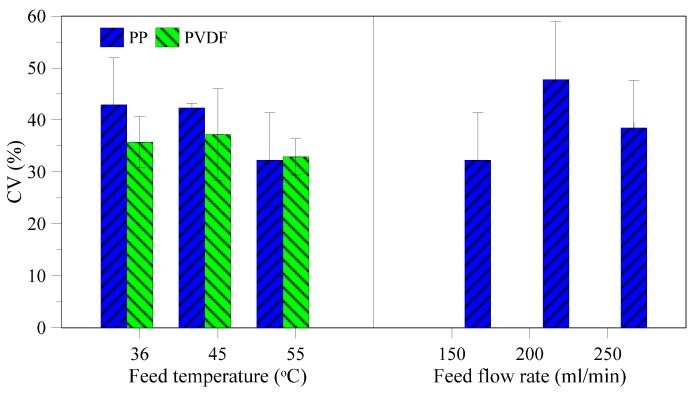
Coefficient of variation (CV) for lab scale modules as function of feed temperature and flow rate.

Coefficient of variation (CV) is a parameter used to characterize the uniformity of the produced crystals. In this study, CV tends to decrease with increasing temperature (Figure 10a). This can be attributed to the easier dissolution of small particles at higher temperatures, thus making the crystal product more uniform with respect to size distribution. No clear tendency is observed for the different feed flowrates as shown in Figure 10b, although the majority of the obtained values show a uniform production by having CV values below 50%, which is normally obtainable for industrial crystallizers [41] and in the ranges of what has been achieved by membrane crystallization in other studies [24].

**Figure 11:**
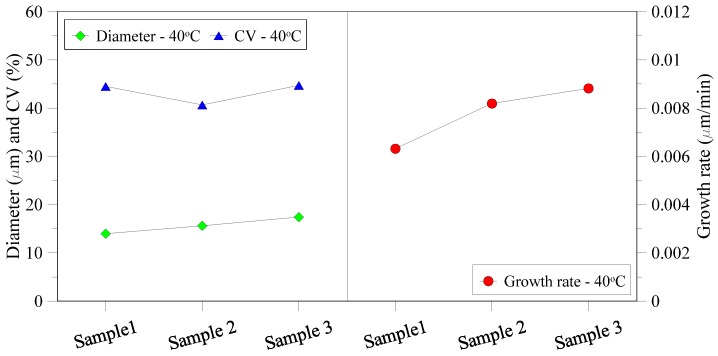
Diameter, coefficient of variance and growth rate for crystals recovered from semi-pilot plant at different time intervals.

Similar to lab scale units, the crystals from semi-pilot scale plant tests were also recovered and characterized. Once the first crystals were observed, a sample of mother liquid containing crystals was analyzed by optical microscopy (Sample 1). Samples 2 and 3 have been characterized after 20 min and 40 min from crystallization onset, respectively. From the crystal images, mean diameter, CV and growth rate were estimated and are shown in Figure 11. Diameter of the crystals is increasing with passage of time due to the nature of crystal growth and the continued incorporation of materials into the crystal lattice. The relative constant increase in diameter and growth rate indicates that the crystallization process is well-controlled and no crystals are growing uncontrollable nor breaking.

**Figure 12:**
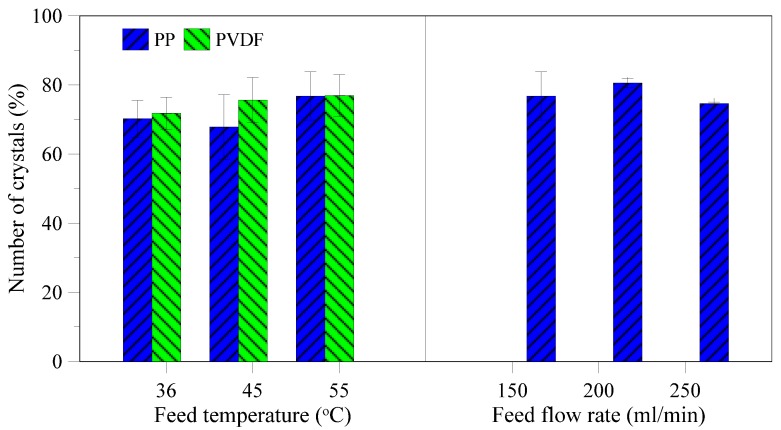
Percentages of analyzed crystals with length to width ratio below 1.4.

Sodium chloride normally has a cubic shape. Deviation from cubic structure can be influenced by the presence of impurities in the solution [24]. The main part of the produced crystals show a length to width ratio below 1.4 (Figure 12), illustrating a good cubic structure. No particular trend between PP and PVDF membrane and the different temperatures and flow rate is observed, thus no disturbance of the cubic crystal growth or incorporation of impurities which can impact the crystal habit has been affected by the various operation conditions (as also proved by the EDX and XRD spectra shown in Figure 6 and Figure 7, respectively).

### 3.3. Membrane Morphology Analysis

Membrane morphology in non-solvent and thermal induced phase separation techniques is a function of operating conditions and dope composition applied at the processing stage and plays a crucial role in dictating overall performance of the membrane distillation process. It was shown in previous studies that membranes containing macrovoids exhibit superior performance against pure water compared with sponge like morphologies [22]. However, the situation can be different when these membranes are applied for treatment of real solutions.

**Figure 13:**
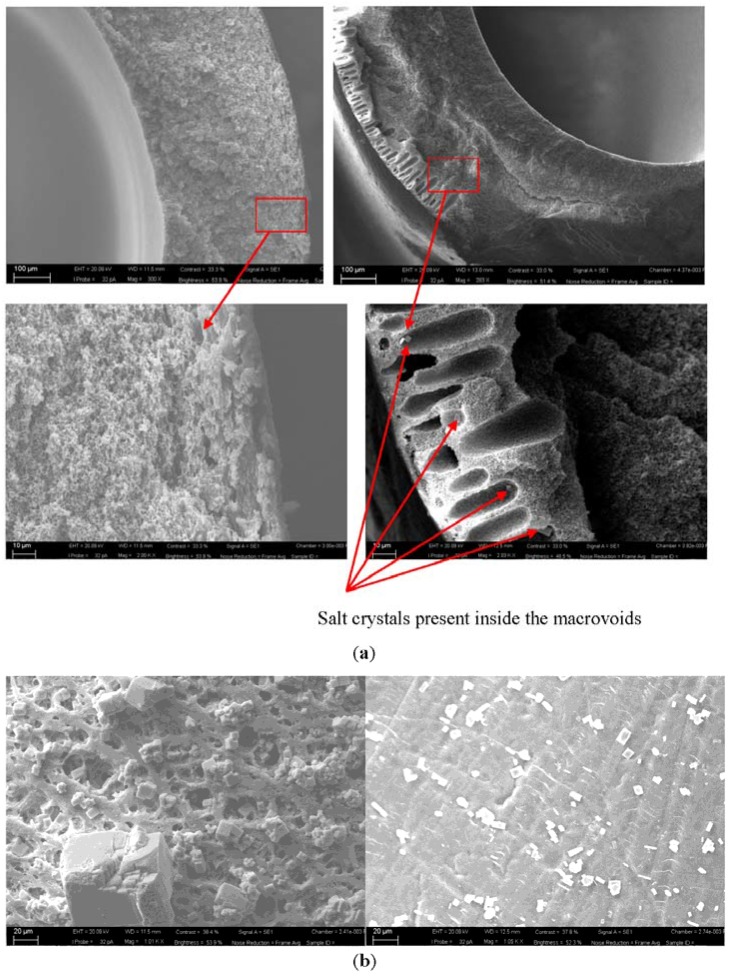
SEM images of PP (**left**) and PVDF (**right**) membranes used (**a**) Images of cross sections of used membrane; salt crystals present in PVDF membrane have been indicated with arrows (**b**) Surface images of the corresponding membranes, crystals within pores and at the surface are evident.

The membranes utilized in the tests were analyzed at the end of the performed experiments. SEM images of the cross sectional morphologies of PVDF and PP membranes used in the current study are shown in Figure 13a,b The figure indicates that the microstructure of PP membrane comprises of sponge like structure whereas the PVDF membranes possess asymmetric structure containing macrovoids on the outer periphery. However, as shown in Figure 13b, the PVDF membrane contains a smooth outer layer whereas in case of PP the outer layer is characterized by the presence of cavity-like structures. Such morphology can be interesting to induce local turbulence at the membrane surface thus improving temperature distribution, however, these can also make severe the scale formation phenomenon at the membrane surface.

Figure 13a shows the cross sectional images of two used membranes and indicates the presence of some salt crystals inside the macrovoids. This is a clear indication of the wetting of macrovoid. On contrary, no salt crystals were detected inside the PP membrane structure that indicates no wetting in case of PP. However, scenario is different for surface scaling where significantly more scales are observed at PP surface. This observation can be related with the surface characteristics of the two membranes investigated. The cavity type structures present at PP membrane acts as anchoring point for crystals to grow and adhere. As illustrated in the figure, it seems that crystals are trapped within the cavities. However, it is not clear from this study whether the crystal growth has started at the surface or in the bulk feed followed by adherence with the surface during the feed circulation along the fiber. In contrast, the smooth surface skin layer of the PVDF membrane does not provide favorable conditions for the crystals to adhere and grow at the surface and, consequently, very little surface scaling has occurred in this case. These observations are coherent with the opinion established by Gryta [21]. The author applied PP membranes with different characteristics and noted that the membranes with large surface pores facilitate the deposition of crystals inside the pores.

The obtained results furnish a useful indication for the selection of appropriate membrane and operative conditions for efficiently carrying out the MCr process, as well as of the necessity to periodically clean the utilized membranes in order to restore their initial performance, thus avoiding wetting and scaling problems.

### 3.4. Cost Analysis

In this work, an economical evaluation of the described process was carried out. The cost analysis was performed considering a 100 m^3^/h plant equipped with pre-filtration for the pre-treatment of the produced waters. All the details of the economical evaluation can be found in [23].

**Figure 14:**
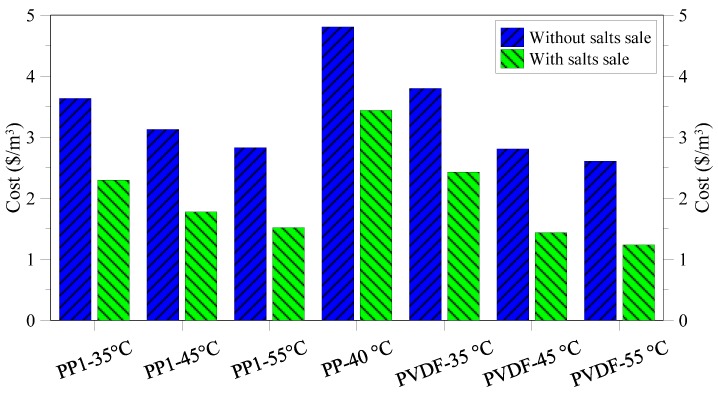
Treatment cost for various operating conditions and membranes applied.

The cost calculation is given in Figure 14 considering performance and properties of the PP and PVDF membrane used in the experiments. For each tested membrane, two different costs were estimated: (i) unit desalted water cost without considering salts sale and (ii) unit desalted water cost with salts sale.

**Figure 15:**
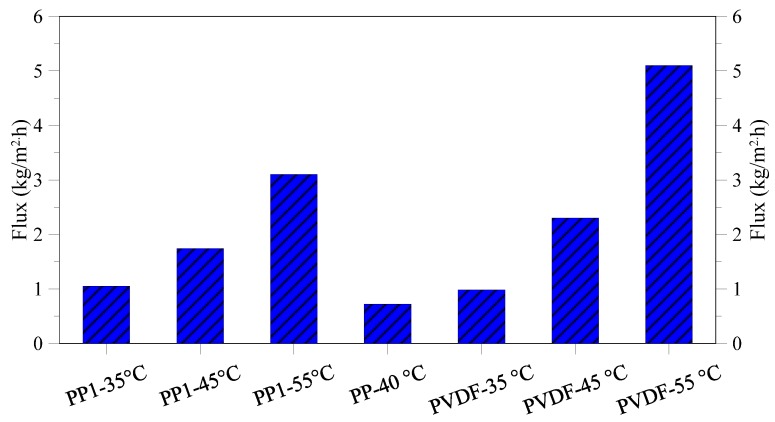
Flux of various membranes tested under different feed inlet temperatures.

The performed economic evaluation indicates that the water unit cost ranges from 4.81 $/m^3^ for PP commercial membrane without considering the gain for the salts sale to 1.24 $/m^3^ for PVDF membrane working at 55 °C. The achieved results strongly depend on the performance of the three different utilized membranes (commercial PP, lab made PP, lab made PVDF) when used in MCr operation. As a matter of fact, the higher the flux (Figure 15), the lower the required membrane area will be and, as a consequence, the membrane and water costs.

It was also observed that, for the same membrane, increasing the operating temperature from 35 to 55 °C, caused an enhancement of trans-membrane flux (thus decreasing the membranes cost both for what concerns capital and operating cost) but also a rise in thermal energy consumption (thus increasing heating cost). However, at least for the results achieved in the tests, the positive effect of the growth of membrane flux (due to the improvement of driving force) completely overcomes the negative effect related to the increase in steam cost. Without considering the salts sale, the minimum cost was 2.83 $/m^3^ and 2.61 $/m^3^ for PP and PVDF lab made membranes, respectively. Considering the high quality of the obtained NaCl salts, the water cost drops to 1.52 $/m^3^ and 1.24 $/m^3^ for PP and PVDF lab made membranes respectively, when the salts sale is taken into account in the economical evaluation.

## 4. Conclusions

In the present work, MCr was applied for the treatment of produced water with the aim to recover the minerals contained. The process showed stable performance when the formed crystals are recovered from the solution. However, at the utilized operating conditions, a decrease in flux was observed for increasing solution concentration due to the suppression of the vapor pressure of the solution. Trans-membrane flux of the system was simulated by using existing heat transfer and vapor pressure depreciation correlations. High quality crystals were produced starting from the water recovery factor of 33%. The crystals showed uniform size distribution (proved by low coefficient of variations) and a good cubic structure. Crystal growth increases by increasing feed temperature due to the higher super-saturation ratio caused by the higher flux. The opposite was the trend of crystal growth with respect to feed flow rates, where the growth decreases with increase of flow rates. Therefore, the limiting step of crystal growth is the integration of material from the interface into the crystal lattice under operative conditions applied in current study. Cavity like structures present at PP membrane act as anchoring points for crystals to adhere and attach with. On the contrary, smooth morphology of the outer surface of the PVDF membrane is more resistant to scale formation. These observations provide a useful insight for the selection of appropriate membrane features for MCr applications in general.

## Symbols and Abbreviations

B	Membrane characteristics parameter
CV	Coefficient of variation (%)
D	Fiber inner diameter (m)
F	Cumulative percent function (µm)
G	Crystal growth rate (µm/min)
h	Heat transfer coefficient (w/K^.^m^2^)
J	Flux (kg/m^2^·s)
L	Crystal length (µm)
M	Molecular weight (kg/mol)
Nu	Nusselts number
*n*^0^	Population density at length zero
P	Vapor pressure (Pa)
R	Resistance (Pa s m^2^/kg)
RF	Recovery factor (%)
T	Temperature (K)
TC	Total carbon (ppm)
TDS	Total dissolved solids (ppm)
TE	Thermal efficiency (%)
TOC	Total organic carbon (ppm)
x	Mole fraction
Suffix	
f	Feed
m	Membrane
p	Permeate
s	Solution
Greek symbols	
*δ*_m_	Membrane thickness (m)
*ε*	Porosity (%)
r	Average pore size (m)
*τ*	Tortuosity factor

## References

[1] Igunnu E.T., Chen G.Z. (2012). Produced water treatment technologies. Int. J. Low-Carbon Technol..

[2] Fakhru’l-Razi A., Pendashteh A., Abdullah L.C., Biak D.R.A., Madaeni S.S., Abidin Z.Z. (2009). Review of technologies for oil and gas produced water treatment. J. Hazard. Mater..

[3] Tellez G.T., Nirmalakhandan N., Gardea-Torresdey J.L. (2002). Performance evaluation of an activated sludge system for removing petroleum hydrocarbons from oilfield produced water. Adv. Environ. Res..

[4] Dahm K., Guerra K. Produced Water Management for Oil and Gas Operations Bureau of Reclamation. Proceedings of the 19th International Petroleum Environmental Conference.

[5] Çakmakce M., Kayaalp N., Koyuncu I. (2008). Desalination of produced water from oil production fields by membrane processes. Desalination.

[6] Kose B., Ozgun H., Ersahin M.E., Dizge N., Koseoglu-Imer D.Y., Atay B., Kaya R., Altınbas M., Sayılı S., Hoshan P. (2008). Performance evaluation of a submerged membrane bioreactor for the treatment of brackish oil and natural gas field produced water. Desalination.

[7] Mondal S., Wickramasinghe S.R. (2008). Produced water treatment by nanofiltration and reverse osmosis membranes. J. Membr. Sci..

[8] Sharghi E.A., Bonakdarpour B., Roustazade P., Amoozegar M.A., Rabbani A.R. (2013). The biological treatment of high salinity synthetic oilfield produced water in a submerged membrane bioreactor using a halophilic bacterial consortium. J. Chem. Technol. Biotechnol..

[9] Ebrahimi M., Willershausen D., Ashaghi K.S., Engel L., Placido L., Mund P., Bolduan P., Czermak P. (2010). Investigations on the use of different ceramic membranes for efficient oil-field produced water treatment. Desalination.

[10] Simon A.R., Fujioka T., Price W., Nghiem L. (2014). Sodium hydroxide production from sodium carbonate and bicarbonate solutions using membrane electrolysis: A feasibility study. Sep. Purif. Technol..

[11] Xu P., Cath T., Drewes J.E. Novel and Emerging Technologies for Produced Water Treatment. Proceedings of the US EPA Technical Workshops for Hydraulic Fracturing.

[12] Camacho L., Dumée L., Zhang J., Li J., Duke M., Gomez J., Gray S. (2013). Advances in membrane distillation for water desalination and purification applications. Water.

[13] Shaffer D.L., Chavez L.H.A., Ben-Sasson M., Castrillón S.R., Yip N.Y., Elimelech M. (2013). Desalination and reuse of high-salinity shale gas produced water: Drivers, technologies, and future directions. Environ. Sci. Technol..

[14] Singh D., Sirkar K.K. (2012). Desalination of brine and produced water by direct contact membrane distillation at high temperatures and pressures. J. Membr. Sci..

[15] Alkhudhiri A., Darwish N., Hilal N. (2013). Produced water treatment: Application of air gap membrane distillation. Desalination.

[16] Curcio E., Criscuoli A., Drioli E. (2001). Membrane crystallizers. Ind. Eng. Chem. Res..

[17] Li W., van der Bruggen B., Luis P. (2014). Integration of reverse osmosis and membrane crystallization for sodium sulphate recovery. Chem. Eng. Process. Process Intensif..

[18] Drioli E., Curcio E., Criscuoli A., Di G. (2004). Integrated system for recovery of CaCO_3_, NaCl and MgSO_4_·7H_2_O from nanofiltration retentate. J. Membr. Sci..

[19] Di Profio G., Tucci S., Curcio E., Drioli E. (2007). Controlling polymorphism with membrane-based crystallizers: Application to form I and II of paracetamol. Chem. Mater..

[20] Guillen-Burrieza E., Thomas R., Mansoor B., Johnson D., Hilal N. (2013). Effect of dry-out on the fouling of PVDF and PTFE membranes under conditions simulating intermittent seawater membrane distillation (SWMD). J. Membr. Sci..

[21] Gryta M. (2007). Influence of polypropylene membrane surface porosity on the performance of membrane distillation process. J. Membr. Sci..

[22] Drioli E., Ali A., Simone S., Macedonio F., L-Jlil S.A.A., al Shabonah F.S., Al-Romaih H.S., Al-Harbi O., Figoli A., Criscuoli A. (2013). Novel PVDF hollow fiber membranes for vacuum and direct contact membrane distillation applications. Sep. Purif. Technol..

[23] Macedonio F., Ali A., Poerio T., El-sayed E., Drioli E., Abdel-Jawad M. (2014). Direct contact membrane distillation for treatment of oilfield produced water. Sep. Purif. Technol..

[24] Macedonio F., Quist-Jensen C.A., Al-Harbi O., Alromaih H., Al-Jlil S.A., al Shabouna F., Drioli E. (2013). Thermodynamic modeling of brine and its use in membrane crystallizer. Desalination.

[25] Khayet M. (2011). Membranes and theoretical modeling of membrane distillation: A review. Adv. Colloid Interface Sci..

[26] Alkhudhiri A., Darwish N., Hilal N. (2012). Membrane distillation: A comprehensive review. Desalination.

[27] Sieder E.N., Tate G. (1936). Heat transfer and pressure drop of liquid in tubes. Ind. Eng. Chem..

[28] Gryta M., Tomaszewska M., Morawski A.W. (1997). Membrane distillation with laminar flow. Sep. Purif. Technol..

[29] Gryta M., Tomaszewska M. (1998). Heat transport in the membrane distillation process. J. Membr. Sci..

[30] Thomas L. (1992). Heat Transfer.

[31] Ali A., Aimar P., Drioli E. (2015). Effect of module design and flow patterns on performance of membrane distillation process. Chem. Eng. J..

[32] Yun Y., Ma R., Zhang W., Fane A.G., Li J. (2006). Direct contact membrane distillation mechanism for high concentration NaCl solutions. Desalination.

[33] Ali A., Macedonio F., Drioli E., Aljlil S., Alharbi O.A. (2013). Experimental and theoretical evaluation of temperature polarization phenomenon in direct contact membrane distillation. Chem. Eng. Res. Des..

[34] Martinez-Diez L., Vazquez-Gonzalez M.I., Florido-Diaz F.J. (1998). Study of membrane distillation using channel spacers. J. Membr. Sci..

[35] Ali A., Quist-Jensen C.A., Macedonio F., Drioli E. Optimization of module length and membrane thickness for membrane distillation. Proceedings of the 2nd International Workshop on Membrane Distillation and Innovating Membrane Operations in Desalination and Water Reuse.

[36] Lawson K.W., Lloyd D.R. (1997). Membrane distillation. J. Membr. Sci..

[37] Phattaranawik J., Jiraratananon R., Fane A. (2003). Heat transport and membrane distillation coefficients in direct contact membrane distillation. J. Membr. Sci..

[38] MartõÂnez-DõÂez M., VaÂzquez-GonzaÂlez L. (1999). Temperature and concentration polarization in membrane distillation of aqueous salt solutions. J. Membr. Sci..

[39] Wilcox W.R. (1972). Crystallization flow. J. Cryst. Growth.

[40] Mersmann A., Eble A., Heyer C. (2001). Crystal growth. Crystallization Technology Handbook.

[41] Myerson A. (2002). Handbook of Industrial Crystallization.

